# Systematic Multi‐Trait Study of Genetic Correlation and Causality Relationships Between General Medical Conditions and Mental Disorders

**DOI:** 10.1111/acps.13825

**Published:** 2025-05-25

**Authors:** Ron Nudel, Maria Da Re, Michael E. Benros

**Affiliations:** ^1^ Copenhagen Research Center for Biological and Precision Psychiatry, Mental Health Center Copenhagen Copenhagen University Hospital Copenhagen Denmark; ^2^ CORE—Copenhagen Research Center for Mental Health, Mental Health Center Copenhagen Copenhagen University Hospital Copenhagen Denmark; ^3^ Psychiatry Unit, Department of Medicine (DAME) University of Udine Udine Italy; ^4^ Department of Clinical Medicine, Faculty of Health and Medical Sciences University of Copenhagen Copenhagen Denmark

**Keywords:** ADHD, depression, obsessive–compulsive disorder, PTSD, schizophrenia

## Abstract

**Introduction:**

Increasing evidence has highlighted bidirectional associations between mental disorders and general medical conditions, with underlying causes ranging from lifestyle habits and side effects from medications to genetic contributions. Novel methods now provide a way to estimate the shared genetic underpinnings and the possibility of a causal relationship between conditions.

**Methods:**

Using summary statistics from large genome‐wide association studies of 16 categories of general medical conditions and 12 categories of mental disorders, we estimated pairwise genetic correlations between general medical conditions and mental disorders using LD score regression. For conditions with significant, positive genetic correlations, we used the latent causal variable (LCV) model to assess the evidence for a causal relationship between them.

**Results:**

Ninety‐five out of 192 pairs of conditions were significantly genetically correlated (*q* ≤ 0.05). Strong and significant correlations were found between conditions such as infections and a psychiatric cross‐disorder phenotype (*r*_g_ = 0.50, *p* = 1.33 × 10^−6^) and irritable bowel syndrome and depression (*r*_g_ = 0.58, *p* = 1.50 × 10^−16^). In the causality analyses, statistically significant evidence for causality was obtained for seven pairs of conditions, including infections being causal to the psychiatric cross‐disorder phenotype, metabolic disorders being causal to attention deficit/hyperactivity disorder (ADHD), post‐traumatic stress disorder (PTSD) being causal to bone and cartilage disorders, arthropathies and epilepsy, obsessive–compulsive disorder (OCD) being causal to irritable bowel syndrome (IBS), and ADHD being causal to arthropathies.

**Conclusions:**

Multiple pairs of general medical conditions and mental disorders were significantly genetically correlated, and for some pairs, there was genetic evidence for a causal relationship. Our findings can inform further molecular studies and clinical practice, raising awareness of the possible co‐occurrence of these conditions.

**Summary**

- Significant outcomes
  ○ The study investigated a large number of general medical conditions and mental disorders, amounting to 192 pairwise comparisons.
  ○ Multiple pairs of general medical conditions and mental disorders are significantly genetically correlated, with the majority of these correlations (94%) being positive.
  ○ Seven pairs of general medical conditions and mental disorders show significant evidence of genetic causality.
- Limitations
  ○ For some phenotypes, a low, albeit significant, heritability estimate was obtained, which could affect downstream genetic correlation and causality analyses.
  ○ Some of the phenotypes included multiple diagnoses, leading to heterogeneity, which could affect the interpretability of the results for these phenotypes.
  ○ Within‐European population differences may affect the estimates of heritability and genetic correlation with the method employed in the study.

## 1 Introduction

Epidemiological studies have shown that numerous general medical conditions are associated with an increased risk of subsequent mental disorders [1, 2, 3], but also that this association is bidirectional, with individuals suffering from mental disorders being at subsequent increased risk of other general medical conditions, and vice versa [4, 5]. Studying the co‐occurrence of somatic and psychiatric conditions is important for the search for the etiological underpinnings of mental disorders and, not least, because general medical conditions substantially contribute to a decreased quality of life, reduced lifespan, and excess mortality in people suffering from mental disorders [6, 7, 8]. Moreover, this co‐occurrence suggests the existence of shared underlying molecular features that may predispose individuals to developing more than one condition [9]. However, observing the association alone is not sufficient to establish the nature of the relationship: lifestyle factors, side effects from medications, and reduced healthcare access have all been highlighted as possible explanations for this phenomenon [7], but other potential mechanisms, such as shared genetic risk [10, 11], have also been proposed, adding to the complexity of these relationships.

Genetic liability of complex phenotypic traits in humans (such as mental disorders, behavioral traits, and many general medical conditions) is known to be highly polygenic and pleiotropic [12], with a significant heritability for mental disorders [13] and a substantial genetic overlap between different disorders [14, 15]. For example, a polygenic risk score for schizophrenia has been shown to be positively associated with digestive problems, respiratory disorders, pregnancy complications, and overall poorer health [10]. A genome‐wide association study (GWAS) found significant pleiotropy between post‐traumatic stress disorder (PTSD) and rheumatoid arthritis, suggesting that similar kinds of immune dysregulation could underlie both conditions [16]. However, even when a clear genetic correlation was found, such as in the case of major depressive disorder (MDD) and cardiovascular disease (CVD) [17], this may reflect two possible scenarios: horizontal pleiotropy, that is, the same loci affecting two different traits indepedently; or vertical pleiotropy, that is, genetic loci affecting Trait 1 which, in turn, leads to Trait 2, thus implying genetic causality [18]. Unlike Mendelian randomization (MR), the latent causal variable (LCV) model is unbiased by pleiotropy and is therefore able to distinguish between a causal relationship and pure horizontal pleiotropy [18, 19]. To our knowledge, LCV model analysis has only been applied in the context of mental disorders to test the relationship between specific pairs of traits [20, 21, 22, 23, 24, 25, 26, 27, 28, 29, 30], but a broad investigation of causality between all major mental disorders and general medical conditions has not yet been conducted.

### 1.1 Aims of the Study

The aim of our study was twofold: firstly, we wanted to provide a comprehensive atlas of genetic correlations between broad categories of general medical conditions, on the one hand, and major mental disorders, on the other hand. Secondly, for pairs of positively genetically correlated conditions, we wanted to assess the presence and direction of causality. The results of these analyses could provide a direction for future investigations that aim to clarify the biological association between different disorders, helping us to understand underlying molecular mechanisms better. In addition, these findings could pave the way for new research on therapeutic targets and preventive strategies, enabling a more personalized approach and improving the quality of life for individuals affected by concurrent disorders.

## 2 Methods and Materials

### 2.1 Selection of Phenotypes

This study was designed to provide an overview of the genetic relationship (be it only correlation or, possibly, causality) between general medical conditions and mental disorders that could potentially have some degree of shared genetic liability. Therefore, we aimed to include a broad range of psychiatric and neurodevelopmental disorders in our study. We selected the main disorders for which summary statistics had been made available on the Psychiatric Genomics Consortium (PGC) website, including studies that used The Lundbeck Foundation Initiative for Integrative Psychiatric Research (iPSYCH) sample, studies that used the UK Biobank, or a combination of these. We covered a broad spectrum of psychiatric dimensions, including schizophrenia, affective disorders (both bipolar disorder and depression), anxiety‐related disorders (such as anxiety and PTSD), eating disorders, neurodevelopmental conditions, and sleep disturbances. In one case (developmental disorders of speech, language, reading, and scholastic skills), we also used summary statistics from FinnGen, which was otherwise the source for the majority of the summary statistics for the general medical conditions in our study. The FinnGen project has been collecting genetic and medical data from the Finnish population using several different biobanks, universities, and hospitals across Finland since 2017. FinnGen makes summary statistics available for a wide range of phenotypes that they have predefined and which are included, in some cases, as broad categories. Whether to include a broad category encompassing multiple diagnoses or a single diagnosis was based on an evaluation taking into account: (1) the epidemiological importance of the condition (e.g., type 2 diabetes mellitus and irritable bowel syndrome (IBS) were evaluated individually); (2) the preexisting degree of evidence linking the two types of conditions (e.g., cancer was included as a broad category due to a lack of evidence linking specific kinds of cancer to specific mental disorders); (3) the need to keep the number of phenotypes analyzed small for statistical reasons (e.g., we included cardiovascular disorders as a single category, considering the fact that cardiovascular alterations frequently co‐occur). For general medical conditions, we performed a literature survey for associations with mental disorders, starting from each individual system (e.g., cardiovascular, digestive, respiratory), and, given the high overlap (genetic or otherwise) between different mental disorders, we chose an inclusive approach, where a general medical condition was tested for genetic correlation with all mental disorders, as long as there was evidence for its association with at least one mental disorder. Evidence supporting the inclusion of general medical conditions is summarized in Table 1. FinnGen summary statistics are from Release 9. Since FinnGen did not include an overall infection phenotype in Release 9, we used summary statistics for overall infection from an iPSYCH study [30], which was a secondary phenotype in that study; the GWAS for overall infection used here did not have a covariate for psychiatric diagnosis, as this is likely to underestimate the former's genetic correlation [54] with the latter or similar phenotypes, but we perform a sensitivity analysis for the inclusion of this covariate in the GWAS for overall infection, for its association with the psychiatric cross‐disorder phenotype (see Supporting Information). Table 2 includes the descriptions of all phenotypes and their sample sizes. References for specific studies from which each summary statistics were derived are also included. ICD‐10 codes [66] for FinnGen phenotypes are provided in Table S1. It is important to note that not all conditions are grouped according to the organ they affect; this is because some conditions are grouped according to etiopathology, and conditions that are purely acquired have not been included in our genetic analysis; for example, autoimmune skin conditions would be included in the “autoimmune diseases” category.

**TABLE 1:** Literature review of the previous associations between the included general medical conditions and mental disorders.

General medical condition	Rationale for inclusion
Cardiovascular diseases	Evidence of genetic correlation between CVD and MDD [17] Increased risk of CVD after a diagnosis of mood disorder [4]
Metabolic disorders	Evidence of genetic correlation between metabolic traits and MDD [17] Pleiotropic action of genes on insomnia and metabolic traits [31]
Malignant neoplasms	Increased risk of cancer after certain psychiatric diagnoses [4] New‐onset psychiatric disorders in older adults linked to increased IRR of cancer [32]
Autoimmune disorders	Association between autoimmune and mood disorders in a youth cohort [9]; increased risk of mood disorders following an autoimmune disorder diagnosis [33] Evidence of genetic overlap between PTSD and some autoimmune disorders [16]; increased risk of autoimmune disorders after a diagnosis of PTSD [34] Bidirectional association between autoimmunity and psychosis [35]; genetic overlap between SCZ and immune functions [36]
Type 2 diabetes	Evidence of genetic correlation between T2D and MDD [17] Genetic overlap between insulin‐related and multiple neuropsychiatric disorders [37]
Infections	Evidence of genetic correlation between infections and psychiatric diagnoses [38] Increased risk of mood disorders following infections in a nationwide cohort [33]
Arthropathies	Negative correlation between the PRS for SCZ and musculoskeletal conditions [10]; decreased risk of musculoskeletal disorders after a diagnosis of SCZ [4] Genetic overlap between PTSD and rheumatoid arthritis [16]
Bone and cartilage disorders	Evidence of decreased bone mineral density in psychiatric patients with unclear relationship to medication status and lifestyle [39, 40] Negative correlation between the PRS for SCZ and musculoskeletal conditions [10]; decreased risk of musculoskeletal disorders after a diagnosis of SCZ [4]
Chronic lower respiratory diseases	Positive correlation between the PRS for SCZ and respiratory disorders [10] Evidence of increased prevalence of emphysema in psychiatric patients regardless of smoking [41] Evidence of genetic correlation between asthma and ADHD, anxiety, and MDD [42]
Organic mental disorders	Increased risk of dementia for people with various psychiatric disorders [43]; depression is a risk factor for any type of dementia [2]
Epilepsy	Preexisting psychiatric disorders associated with new‐onset epilepsy in adults [44] Evidence of genetic correlation between SCZ and epilepsy [45] Evidence of genetic correlation between ADHD and epilepsy [46]
Inflammatory bowel diseases	Positive correlation between the PRS for SCZ and digestive disorders [10]. Positive correlation between a PRS for IBD and psychiatric comorbidity [47] Higher risk of IBD in veterans after a diagnosis of PTSD [34] Increased risk and higher prevalence of psychiatric disorders and suicidality in people with IBD [48, 49]
Irritable bowel syndrome	Evidence of genetic overlap between IBS and psychiatric disorders [50] Positive correlation between the PRS for SCZ and digestive disorders [10]
Spontaneous abortion	Positive correlation between the PRS for SCZ and complications of pregnancy, childbirth, and puerperium [10] Evidence of genetic correlation between miscarriage and depressive symptoms, anxiety, and insomnia [51]
Pre‐ or eclampsia	Positive correlation between the PRS for SCZ and complications of pregnancy, childbirth, and puerperium [10] Increased risk of postpartum psychiatric first episodes following pre‐eclampsia [52]
Complications of labor and delivery	Positive correlation between the PRS for SCZ and complications of pregnancy, childbirth and puerperium [10] Increased risk of labor and delivery complications in women with psychosis [53]

Abbreviations: ADHD: attention deficit/hyperactivity disorder; CVD: cardiovascular disease; IBD: inflammatory bowel disease; IBS: irritable bowel syndrome; IRR: incidental relative risk; MDD: major depressive disorder; PRS: polygenic risk score; PTSD: post‐traumatic stress disorder; SCZ: schizophrenia; T2D: type‐2 diabetes.

**TABLE 2:** Phenotype description including sample sizes from the included studies.

Phenotype	Description of cases	Sample size^a^	Reference
Schizophrenia	Schizophrenia	130,644	[55]
Autism spectrum disorder	Autism spectrum disorder	46,350	[56]
Attention deficit/hyperactivity disorder	Attention deficit/hyperactivity disorder	225,534	[57]
Depression	MDD and broad‐definition depression	500,199	[58]
Bipolar disorder	Bipolar disorder type I and type II	413,466	[59]
Post‐traumatic stress disorder	Formal diagnosis of PTSD and self‐reported PTSD symptoms in people exposed to traumatic events	174,659	[60]
Anxiety disorders	GAD, panic disorder, agoraphobia, social phobia, specific phobias	17,310	[61]
Obsessive–compulsive disorder	Obsessive–compulsive disorder	9725	[62]
Anorexia nervosa	Anorexia nervosa restricting or binge‐purge subtype, eating disorder NOS—anorexia nervosa subtype (according to DSM‐IV)	72,517	[63]
Insomnia	Anyone who received a diagnosis of insomnia or was treated for insomnia	386,988	[31]
Developmental language, speech, reading, and scholastic skills disorders	Specific developmental disorders of speech and language, reading, and scholastic skills. Note for controls: *excluding* any other psychiatric diagnosis	280,097	[64]
Psychiatric cross‐disorder	ADHD, ASD, schizophrenia, depression, bipolar disorder, and anorexia nervosa	65,534	[65]
Cardiovascular diseases	All cardiovascular disorders *including* cerebrovascular and venous system diseases *excl*. cardiac rheumatic disease	377,277	[64]
Metabolic disorders	Disorders involving the metabolism of amino acids, lipids, carbohydrates, ions, fluid, and acid–base balance, both congenital and acquired *including* lactose intolerance, cystic fibrosis, hypercholesterolemia, amyloidosis, *excluding* diabetes mellitus	377,277	[64]
Malignant neoplasms	All malignant neoplasms	364,462	[64]
Autoimmune disorders	All disorders of autoimmune origin, *including* autoimmune thyroiditis, coeliac disease, type 1 diabetes mellitus, and multiple sclerosis	377,277	[64]
Type 2 diabetes	Non‐insulin‐dependent diabetes, with or without complications	365,950	[64]
Infections	Any infection, encompassing categories such as central nervous system infections, gastrointestinal infections, respiratory infections, skin infections, sepsis, otitis, hepatitis, urological infections, genital infections, and pregnancy‐related infections, HIV infection, as well as broad categories of bacterial, viral, and parasitic infections	65,534	[30]
Arthropathies	All diseases primarily affecting joints *excluding* acute traumatic injury	377,277	[64]
Bone and cartilage disorders	All disorders primarily affecting bone and cartilage *including* stress fracture, pathological fracture *excluding* acute traumatic injury	377,277	[64]
Chronic lower respiratory diseases	All chronic lower respiratory diseases, both infectious and non‐infectious	377,277	[64]
Organic mental disorders	Any cause of dementia, postencephalitic syndrome, psychiatric, and behavioral symptoms due to organic conditions, delirium	373,159	[64]
Epilepsy	Any type of epilepsy *excluding* status epilepticus, Landau–Kleffner syndrome	299,577	[64]
Inflammatory bowel diseases	Ulcerative colitis and Crohn's disease	377,277	[64]
Irritable bowel syndrome	Irritable bowel syndrome, with or without diarrhea	311,254	[64]
Spontaneous abortion	Any case of spontaneous abortion	166,528	[64]
Pre‐ or eclampsia	Pre‐eclampsia, pre‐eclampsia on chronic hypertension, HELLP syndrome, eclampsia	201,478	[64]
Complications of labor and delivery	Any complication of labor and delivery	210,870	[64]

Abbreviations: ADHD: attention deficit/hyperactivity disorder; ASD: autism spectrum disorder; DSM‐IV: diagnostic and statistical manual of mental disorders 4th edition; GAD: generalized anxiety disorder; HELLP: hemolysis, elevated liver enzymes, and low platelets; MDD: major depressive disorder; NOS: not otherwise specified; PTSD: post‐traumatic stress disorder.

^a^ The sample sizes are from the documentation supplied with the summary statistics or from the relevant article.

### 2.2 Data Cleaning and Processing

Summary statistics were obtained from the aforementioned sources. All summary statistics were loaded into R v4.3.0 [67] and the variables needed for downstream analysis with LDSC and LCV were checked. In cases in which the sample size for each marker was not specified, we calculated the total sample size based on counts for cases and controls included in the summary statistics, where available, or, otherwise, based on a constant sample size from the documentation provided with the summary statistics or from the publication. The effect allele was identified based on the documentation or based on the publication, in cases in which no documentation had been provided. Otherwise, “A1” was assumed to be the effect allele based on convention. FinnGen summary statistics were filtered to exclude rows with markers without rsIDs. For iPSYCH summary statistics, marker IDs were converted to rsIDs using the HRC reference (ftp://ngs.sanger.ac.uk/production/hrc/HRC.r1‐1/HRC.r1‐1.GRCh37.wgs.mac5.sites.tab.gz) from the same genome build (hg19), based on chromosome and position, prior to downstream analysis. Next, all summary statistics datasets were processed with the LDSC [14, 68] v1.0.1 *munge_sumstats.py* script. Using the *‐‐merge‐alleles* option, markers were retained if they were present in a list of high‐quality markers (https://data.broadinstitute.org/alkesgroup/LDSCORE/w_hm3.snplist.bz2), and markers with mismatched alleles as compared to this reference were removed.

### 2.3 Estimation of Heritability and Genetic Correlation

LDSC was used to estimate the heritabilities (*‐‐h*2) and the genetic correlations (*‐‐rg*) for phenotypes in our study. The LD score dataset was the European dataset from the LDSC website (https://data.broadinstitute.org/alkesgroup/LDSCORE/eur_w_ld_chr.tar.bz2), which was used as both the reference and weight LD score dataset, as recommended in the tutorial for non‐partitioned regression. *p* values were calculated from the *χ* ^2^ distribution with one degree of freedom, using a Wald statistic (calculated as the square of the quotient of the heritability or the genetic correlation estimate divided by their respective standard error, as provided by LDSC). *p* values for heritability estimates were multiplied by 0.5 (testing whether they were significantly above zero), while *p* values for genetic correlation estimates were not (testing whether they were significantly different from zero). Heritability estimates were performed in order to test for the presence of a polygenic signal for the phenotype; therefore, phenotypes were included in downstream analyses if their heritabilities were at least nominally significantly above zero (*p* ≤ 0.05). Genetic correlation analyses were performed for every possible somatic–psychiatric phenotype pair. Once all pairwise genetic correlations were tested, we used a false‐discovery rate (FDR)‐based correction method to calculate *q* values for all genetic correlation *p* values with QVALUE v2.32.0 [69] with the default parameters. Defining significant associations using a *q* value threshold of *α* means the expected proportion of false positives among this subset of (significant) associations is *α* [70]. The corrplot package v0.92 [71] was used to generate a correlation plot.

### 2.4 Causality Analysis

For every positive genetic correlation with *q* ≤ 0.05, we performed an LCV analysis [19] using the following scripts: https://github.com/lukejoconnor/LCV/blob/master/R/ExampleRealdataScript.R (updated July 1, 2020), https://github.com/lukejoconnor/LCV/blob/master/R/MomentFunctions.R (updated March 14, 2019), and https://github.com/lukejoconnor/LCV/blob/master/R/RunLCV.R (updated March 14, 2019). In this study, we focused on pairs of conditions that were significantly positively genetically correlated (which made up the vast majority of pairs), as the focus of our study was on the translational value of the results; if a causality relationship between two conditions can be shown, then treating one could improve the outcome of the other. The LCV model assumes a latent causal variable, L, which mediates the genetic correlation between Trait 1 and Trait 2; if Trait 1 is strongly correlated with this variable, then part of the genetic component of Trait 1 is causal to Trait 2, and this partial genetic causality is quantified using the genetic causality proportion (GCP) statistic (the LCV program provides a two‐sided *p* value for the GCP estimate being different from zero). The GCP is interpreted in the following manner: a GCP value of 0 or a nonsignificant GCP does not provide evidence for a causal relationship between the two traits; a significant GCP in the range 0 < GCP ≤ 1 suggests that Trait 1 is causal to Trait 2, and a significant GCP in the range −1 ≤ GCP < 0 suggests that Trait 2 is causal to Trait 1. It is therefore important to note which phenotype was defined as Trait 1 and which phenotype was defined as Trait 2. The strength of evidence for the causal relationship can be inferred from the absolute value of the GCP, that is, |GCP|, where a value of 1 indicates full genetic causality (0.6 was the cutoff value for indicating strong genetic causality in the LCV method paper). We chose to use the LCV model for analyses for the following reasons: LCV has weaker assumptions than traditional MR methods; LCV uses genome‐wide data without the need to preselect specific SNPs (which otherwise must be shown to be associated with the exposure trait); LCV does not require defining which trait is the exposure and which is the outcome (the sign of the GCP indicates the direction of the relationship); LCV produces fewer false positives in the presence of genetic correlation than MR [19]. This approach thus allowed us to apply the same procedure to multiple pairs of diseases without the need to make specific assumptions on the direction of the relationships or the included markers. Following the LCV analyses, *q* values were calculated using the above procedure. We used the same LD scores and summary statistics datasets with LCV as we did with LDSC.

### 2.5 Post Hoc Mendelian Randomization Analysis

For causal relationships that were highlighted in the LCV results, and where the exposure phenotype had genome‐wide significant SNPs, we performed an inverse‐variance weighted two‐sample Mendelian randomization (IVW‐MR) using the MRlap R package v0.0.3.3 [72]; this method relies in part on LDSC and can correct the test statistics for different biases. The same processed summary statistics datasets that were used with LDSC and LCV, and the same LD score dataset, were used with this package. The analyses were run with the default parameters.

## 3 Results

We included a total of 28 phenotypes in our analysis, where 16 were broad categories of general medical conditions and 12 major psychiatric and neurodevelopmental disorders. The summary statistics for the psychiatric cross‐disorder phenotype are from a GWAS which included as cases individuals with one or more of six major psychiatric diagnoses: ADHD, ASD, schizophrenia, depression, bipolar disorder, and anorexia, but we note that the article for this GWAS identified significant associations for the cross‐disorder phenotype specifically (see Table 2 for phenotype definitions). Only two somatic phenotypes correspond to specific diagnoses, namely type 2 diabetes (T2D) and IBS.

### 3.1 Heritability (*h* ^2^) Analyses

All 28 phenotypes showed at least nominally significant heritabilities (*p* ≤ 0.05), with schizophrenia, obsessive–compulsive disorder (OCD) and autism spectrum disorder (ASD) showing the highest estimates. Heritability estimates on the observed scale are provided in Table S2.

### 3.2 Genetic Correlation (*r*_g_) Analyses

**FIGURE 1:**
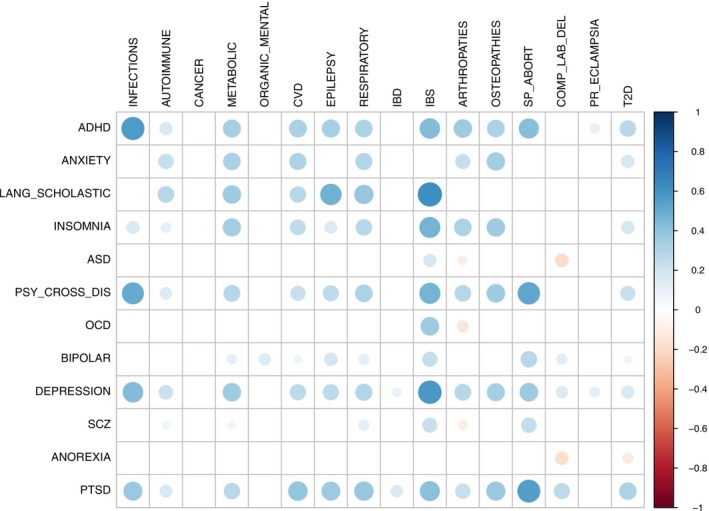
Genetic correlation analyses between somatic diseases and psychiatric disorders. The effect size is indicated by the color of the circle (the absolute value of the effect size is also indicated by the size of the circle). Only correlation estimates surviving FDR correction for multiple testing (*q* ≤ 0.05) are shown. ADHD: attention deficit/hyperactivity disorder; ASD: autism spectrum disorder; COMP_LAB_DEL: complications of labor and delivery; CVD: cardiovascular diseases; IBD: inflammatory bowel diseases; IBS: irritable bowel syndrome; LANG_SCHOLASTIC: developmental language, speech, reading, and scholastic skills disorders; OCD: obsessive–compulsive disorder; ORGANIC_MENTAL: organic mental disorders; PR_ECLAMPSIA: pre‐ or eclampsia; PSY_CROSS_DIS: psychiatric cross‐disorder; PTSD: post‐traumatic stress disorder; SCZ: schizophrenia; SP_ABORT: spontaneous abortion; T2D: type 2 diabetes.

Out of the 192 pairs that were tested, 95 showed a significant genetic correlation (*q* ≤ 0.05). The majority of the correlations were positive, suggesting overall concordant allelic effects between the phenotypes. The largest *r*_g_ among the significantly positively correlated phenotypes was for IBS and developmental disorders of speech, language, reading, and scholastic skills. The results of the genetic correlation analyses for all pairs of general medical conditions and psychiatric and neurodevelopmental disorders are shown in Figure 1. The full results can be found in Table S3.

### 3.3 Causality Analyses

Of the 89 significant and positive genetic correlations that were selected for downstream LCV analysis, 16 obtained a statistically significant GCP estimate (*q* ≤ 0.05). However, some of them were potentially biased due to low *h* ^2^ *z*‐scores and/or potentially nonsignificant *r*_g_s (estimated by LCV rather than by LDSC). When considering results not flagged for either of these issues, we found significant and robust causality relationships for seven pairs of conditions, as shown in Table 3. Two general medical conditions were found to be causal to mental disorders, namely infections being causal to the psychiatric cross‐disorder phenotype and metabolic disorders being causal to attention deficit/hyperactivity disorder (ADHD). In all other cases, the causal relationship indicated mental disorders as causal to general medical conditions. The complete results of the LCV analyses are provided in Table S4.

**TABLE 3:** Significant results from the LCV analysis of causality.

Trait 1	Trait 2	GCP	*p*	*q*
General medical conditions being causal to mental disorders				
Attention deficit/hyperactivity disorder	Metabolic disorders	−0.1623	1.67 × 10^−18^	1.35 × 10^−17^
Psychiatric cross‐disorder	Infections	−0.8310	9.62 × 10^−08^	3.88 × 10^−07^
Mental disorders being causal to general medical conditions				
Obsessive–compulsive disorder	Irritable bowel syndrome	0.0374	1.01 × 10^−63^	3.26 × 10^−62^
Post‐traumatic stress disorder	Bone and cartilage disorders	0.8484	4.22 × 10^−11^	2.27 × 10^−10^
Post‐traumatic stress disorder	Epilepsy	0.6648	0.0001	0.0004
Post‐traumatic stress disorder	Arthropathies	0.3607	0.0075	0.0186
Attention deficit/hyperactivity disorder	Arthropathies	0.2642	0.0189	0.0381

*Note:* A GCP > 0 suggests Trait 1 is genetically causal to Trait 2, and a GCP < 0 suggests that Trait 2 is genetically causal to Trait 1. The results reported in this table met stricter significance criteria (see Methods).

Abbreviation: GCP: genetic causality proportion.

Two post hoc IVW‐MR analyses were performed for pairs of phenotypes from Table 3 in which the exposure phenotype had genome‐wide significant markers (which could be used as instrument variables (IVs)): ADHD being causal to arthropathies and metabolic disorders being causal to ADHD. The former (with 22 IVs) found a significant causal effect for ADHD on arthropathies (corrected effect = 0.109; SE = 0.047, *p* = 0.021), whereas the latter (with 16 IVs) did not (*p* = 0.059).

## 4 Discussion

Our analysis identified significant genetic correlations across various categories of conditions. This is consistent with previous studies showing high degrees of pleiotropy across several somatic and psychiatric domains [16, 17, 45, 73]. It should be noted that our analyses are based on European samples, making them less generalizable. It is also important to consider that the estimate of genetic correlation will be reduced in the presence of both concordant and discordant allelic effects for the two phenotypes across many loci. Therefore, an *r*_g_ close to zero does not necessarily mean that there is no pleiotropy [14]. Also, as LDSC estimates *r*_g_ from an equation that includes *h* ^2^ in the denominator, *r*_g_ might be inflated for diseases with low *h* ^2^ [18], which may be the case for developmental disorders of language, speech, reading, and scholastic skills and IBS. Regarding the former, this could be because the proportion of cases for this phenotype in FinnGen was < 1%, while even for only one of the conditions included in this category, language impairment, the prevalence is 1%–10% [74]. Second, there could be a high degree of heterogeneity within this category.

Notably, some conditions such as cancer and organic mental disorders did not show many or any significant genetic correlations. It may be the case that the epidemiological correlations between these conditions and mental disorders reported in some studies [4, 43] are due to other reasons, such as possible side effects from medications or consequences of lifestyle habits. Alternatively, the psychiatric manifestation could potentially be an early sign of a yet undetected general medical condition such as cancer [32]. Conversely, psychiatric diagnoses such as depression, PTSD, insomnia, ADHD, and psychiatric cross‐disorder show stronger genetic correlations with several general medical conditions. While in the first three cases it may be that the correlation is due to an underlying chronic activation of the stress response mechanisms with subsequent dysregulation of immune‐inflammatory and metabolic functions impacting both physical and mental health, in the case of ADHD, the extensive pattern of correlations is more difficult to interpret, but it is consistent with a recent study showing that genetic liability to multiple complex traits influences the risk of ADHD and vice versa [27]. Interestingly, other highly heritable conditions such as ASD, OCD and anorexia nervosa show little genetic correlation with other medical conditions, with ASD also displaying a different pattern of correlations than shown with ADHD [75]. Lastly, one notable result is the strong positive genetic correlation between epilepsy and developmental disorders of language, speech, reading, and scholastic skills. This result is interesting in light of previous accounts of pleiotropy between language disorders and epilepsy—although these often involved specific loci and/or structural alterations [76, 77]—and it is important, given that cognitive impairment in the context of pediatric epilepsy decreases the quality of life for patients [78].

With regards to the causality analyses, of the seven pairs of disorders that had a significant nonzero GCP with *q* ≤ 0.05, three pairs also showed |GCP| > 0.6 (the cutoff value used in the original LCV paper) [19]. In the case of infections, a strong epidemiological correlation with mental disorders has been reported [33, 38, 79]. Moreover, infections seem to have a time‐related, persistent, and dose‐dependent effect on the risk of subsequent mental disorder, both in terms of total number of infections and number of different infection types [30, 33], regardless of family history of psychiatric illness and substance abuse [33, 79]. Even mild infections may increase the risk for mental disorders [80]. Genetic differences between individuals with ASD born to mothers with or without pregnancy‐related infections may suggest that infections play an independent role as risk factors for ASD [81]. The link between infections and subsequent mental disorders may include activation of immune‐related cells and molecules within the CNS, together with an increased blood–brain barrier permeability, potentially altering neurotransmitter production [82, 83]. It is important to note, however, that the psychiatric cross‐disorder phenotype comprised several disorders with different case sample sizes, and the genetic link between this phenotype and infections might be driven by some of these and not others, as indicated by the different genetic correlation patterns between infections and the individual psychiatric disorders.

With regards to PTSD, studies reported significant pleiotropy between PTSD and inflammatory conditions, even though the identified genes are not always concordant across studies [16, 60]. Epidemiological correlations between PTSD and several autoimmune disorders have been observed [34] as well as a more specific association with rheumatologic disorders such as rheumatoid arthritis, fibromyalgia [84], and chronic pain [85]. Growing evidence is highlighting a dose‐dependent effect of stressful life events on bone mineralization [86] and an increased risk of developing osteoporosis with an earlier onset in people with PTSD [87], and these findings are consistent also with animal model studies [88]. Even though the influence of confounders (e.g., smoking and medication) could only be ruled out with further studies, the disruptive effect of traumatic stress on glucocorticoid production, cytokines, and cellular immunity is well documented [89]. Together with our results, these findings suggest that the role of chronic inflammation and immunological dysregulation occurring in PTSD is a putative etiological factor in disorders of the bone tissue and joints. Concerning the association with epilepsy, an increased prevalence of PTSD among epileptic patients compared to controls and a temporal relationship between PTSD and subsequent onset of epilepsy have been observed, suggesting possible causality at least in a subset of patients [90, 91]. This is consistent with studies showing facilitation in the epileptogenic process following intense stress and depressive states [92, 93], which further supports the idea of a “diathesis‐epilepsy model” [94], possibly mediated by effects of corticosteroids [95]. Nonetheless, the complexity of interactions between epilepsy and PTSD also includes the traumatic potential of epileptic seizures [91] and the existence of psychogenic non‐epileptic seizures as possible manifestations of PTSD with dissociative features [96].

IBS has an epidemiological correlation with anxiety and depression [97], but studies specific to OCD are still limited [98, 99, 100]. IBS could be more similar to mental disorders than to other gastrointestinal conditions [50], suggesting a primary neurobiological alteration that may cause the dysregulation in visceral sensitivity and autonomic system typical of IBS [101].

With regards to metabolic disorders (which include both congenital and acquired deficits of several metabolic pathways, see Table 2) being causal to ADHD, there is some evidence suggesting that dysregulation of some ions is a possible cause of neurodegeneration and neurotoxicity [102], mostly via an increase in oxidative stress, activation of the NMDA receptor and interference in neurotransmission [103]. Previous studies consistently indicate the possible role of electrolyte imbalance in ADHD [27, 104]. Moreover, docosaexahenoic acid (DHA)—which is essential to brain development [105]—may also be relevant, as it is frequently deficient both in ADHD [106] and in metabolic diseases such as cystic fibrosis [107] and phenylketonuria [108]. Concerning arthropathies, it is important to note that an association between ADHD, joint hypermobility and developmental coordination disorder is already known [109, 110, 111], and children and adolescents with ADHD suffer more traumatic musculoskeletal injuries than controls [112]. A previous study, which also used the LCV method, explored potential causality between ADHD and multiple complex traits and reported results suggesting an opposite direction of causality [27]; however, in our study we used updated summary statistics with more cases [57], and this causal effect was also found in a post hoc IVW‐MR analysis. Interestingly, according to another recent study using the LCV method, hyperactive and manic states were found to be causal to osteoarthritis of the hip and knee [113]. A different study confirmed a small but significant genetic correlation between ADHD and rheumatoid arthritis [73]. Further research is needed to disentangle the complex relationships underlying these correlations. It should be noted that, given that the genetic correlation estimate in the LDSC model is standardized by the heritabilities of the phenotypes, and that LCV uses the heritabilities to normalize the summary statistics, low heritabilities may increase the genetic correlation estimate and impact the GCP estimate. In line with the LCV method paper, we interpret the GCP as an indication of the strength of the evidence for a causal genetic relationship and not as an effect size for causality.

### 4.1 Strengths and Limitations

The main strengths of this study are the breadth of the included mental disorders and somatic disease categories and the methodology used, which allowed for consistent processing and application to all phenotypes with a minimum number of assumptions. There are, however, some limitations which should be kept in mind when interpreting the results. As the focus of the study was mental disorders and their relationships with many other types of disorders and diseases, the former were included as specific/narrower categories, while the latter were included as broader categories (with some exceptions). While this allowed us to include many different diagnoses, it does affect the interpretability of the results. It also means that sample sizes for the different diagnoses differed greatly within and across categories, and the differences in the statistical power across the discovery GWASs may also influence the genetic correlation estimates. Also, we cannot rule out that some results for the diseases included in specific GWASs were affected to some degree by “spurious comorbidity bias,” especially in studies from case–control cohorts for diseases with multiple comorbidities [114]. Similarly, it cannot be ruled out that there are nongenetic factors influencing the relationships between conditions, as outlined in the introduction, which were not accounted for in the discovery GWASs; importantly, the discovery GWASs were not adjusted for possible comorbidities between conditions, which could influence the observed association between them. Lastly, we used the FinnGen cohort (from Finland) for many of the phenotypes in our study; while the LDSC method paper did examine the stability of the LD scores across specific European populations, including Finns, and concluded that they were highly correlated, and that their European LD score panel is adequate for populations of northern European ancestry [68], it is known that the Finnish population underwent a genetic bottleneck event resulting in deleterious alleles not common in other European populations [64]. This could lead to some bias in the estimates when analyzing FinnGen summary statistics.

We therefore envision our study as a starting point to further, more specific analyses, which could focus on individual conditions within each category. We did not aim to investigate specific genetic mechanisms underlying the relationships we identified, but rather to use genetic data to infer them. Lastly, with regard to the causality analyses, it should be emphasized that the method we used provides only statistical evidence for genetic causality. Further studies are required to identify the molecular mechanisms which underlie these relationships.

## 5 Conclusions

Our results indicate multiple genetic correlations and potential causal relationships between general medical conditions and mental disorders. While we used broad categories of general medical conditions and genome‐wide approaches, our results could direct future molecular studies examining the role of specific genetic variants in the relationships between highlighted pairs of conditions. In line with the interpretation of the LCV results, in cases of a genetic causal relationship, proper diagnosis and treatment of the causal condition may ameliorate the other condition, depending on the timing of the treatment and the nature of the relationship. In this sense, our study offers important translational value, and we hope that it will raise awareness of these links among healthcare professionals and encourage further investigations into the specific mechanisms underlying these links.

## Author Contributions

R.N. conceived and designed the study, performed quality control and genetic analyses, interpreted the results, and wrote the manuscript. M.D.R. performed quality control and genetic analyses, performed the literature survey, interpreted the results, and wrote the manuscript M.E.B. conceived and supervised the study, interpreted the results, and revised the manuscript.

## Ethics Statement

The authors have nothing to report.

## Consent

The authors have nothing to report.

## Conflicts of Interest

The authors declare no conflicts of interest.

## Supporting information


Data S1.

## Data Availability

This study used summary statistics from previously published studies or online accessible resources.
