# Supplementary material for: Systematic Multi‐Trait Study of Genetic Correlation and Causality Relationships Between General Medical Conditions and Mental Disorders
Source: Acta Psychiatr Scand. 2025 May 25;152(3):236–49. doi: 10.1111/acps.13825 (PMC12318648; doi:10.1111/acps.13825)
Supplement: Supplementary file 1 — Data S1. [file ACPS-152-236-s001.pdf]

Supplementary material for: **Systematic multi-trait study of genetic correlation and causality relationships between general medical conditions and mental disorders**

Table of Contents:

- Supplementary Table S1: ICD-10 codes for FinnGen phenotypes
- Supplementary Table S2: Heritability estimates for all investigated phenotypes
- Supplementary Table S3: Pairwise genetic correlations for all somatic-psychiatric disorder pairs
- Supplementary Table S4: Results of the LCV analyses
- Sensitivity analyses for the infections phenotype

Supplementary Table S1: ICD-10 codes for FinnGen phenotypes

Phenotype	ICD-10 codes for cases	Exclusion criteria for controls (ICD-10 codes) in addition to not being cases	Internet link to the FinnGen phenotype description.
Cardiovascular diseases	I10-I15, I13.2, I20-I27, I30-I42, I42.1, I42.3-I42.7, I44-I48, I50, I60, I61, I63, I64, I66, I67.1, I67.3, I67.4, I67.6, I67.7, I68.1*, I68.1*A18.8, I68.1*A32.8, I68.1*A52.0, I68.1*B02.8, I68.2*, I68.2*M32.1, I70, I71.0, I71.01, I71.09, I71.1-I71.6, I71.8, I71.9, I72, I74, I74.3, I80, I80.20#, I80.29#, I80.3#, I81, I82, I83, I85, I88, I95	None	https://r9.risteys.finngen.fi/endpoints/FG_CVD
Metabolic disorders	E70-E90	None	https://r9.risteys.finngen.fi/endpoints/E4_METABOLIA
Cancer	C00-C26, C30-C34, C37-C41, C43-C58, C60-C86, C88, C90-C97	Any C code	https://r9.risteys.finngen.fi/endpoints/C3_CANCER_EXALLC
Autoimmune disorders	D51, D59#, D69, D69.3, E03.80, E03.82, E03.89, E03.9, E03.09+F0.89, E05.09, E06.03, E10, E27.1-E27.4, E31, E31.08, E31.1	None	https://r9.risteys.finngen.fi/endpoints/AUTOIMMUNE

	G04.0#, G35-G37, G47.4, G61, G70#, G73, H06.2*E05.9, H20, E20.1, I00, I00+J17.8, I00+L54.0, I01, I02, I05-I09<br>K50, K51, K74.3, K75.4, K90, L12, L12.1L12.3, L12.8, L12.9, L13, L40-L40.5, L40.8, L40.9, L63, L80, M05, M06, M30.1, M31, M31.3, M31.7, M32, M32.8, M32.9, M33#, M34, M35, M35.2, M35.9<br>N08.2*D69.0, N08.2*D89.8		
Type 2 diabetes	E11, E11.01, E11.09, E11.2+, E11.2+N08.39, E11.3+, E11.3+H28.0, E11.3+H36.09,<br>E11.4+, E11.4+G59.0, E11.4+G63.2, E11.4+G73.0, E11.4+G99.0, E11.5,<br>E11.6, E11.6+M14.2, E11.6+M14.6, E11.7, E11.8	E10-E13	https://r9.risteys.finngen.fi/endpoints/T2D
Arthropathies	M00-M25	None	https://r9.risteys.finngen.fi/endpoints/M13_ARTHROPATHIES
Bone and cartilage disorders	M80-M94, M90. M80-M94, M90.6*C40.99, M90.6*C41.996*C40.99, M90.6*C41.99	None	https://r9.risteys.finngen.fi/endpoints/M13_OSTEOCHONDRO

Chronic lower respiratory diseases	J40-J47	None	https://r9.risteys.finngen.fi/endpoints/J10_LOWCHRON
Organic mental disorders	F00-F09	F00-F09	https://r9.risteys.finngen.fi/endpoints/F5_DEMENTIA
Epilepsy	G40	G40-G47	https://r9.risteys.finngen.fi/endpoints/G6_EPLEPSY
Inflammatory bowel disease	K50-K51	K50-K51, K83	https://r9.risteys.finngen.fi/endpoints/K11_IBD_STRICT
Irritable bowel syndrome	K58	K55-K64	https://r9.risteys.finngen.fi/endpoints/K11_IBS
Spontaneous abortion (only females)	O03	O00-O08	https://r9.risteys.finngen.fi/endpoints/O15_ABORT_SPONTAN
Pre- or eclampsia (only females)	O11, O14, O15	O10-O16	https://r9.risteys.finngen.fi/endpoints/O15_PRE_OR_ECLAMPSIA
Complications of labour and delivery (only females)	O60-O75	None	https://r9.risteys.finngen.fi/endpoints/O15_COMPLIC_LAB_DELIV
Developmental language, speech, reading and scholastic skills disorders	F80-F81	F00-F09, F05.1, F1-F9, X60-X84, G30	https://r9.risteys.finngen.fi/endpoints/KRA_PSY_SPEELING_EXMORE

Note that in some cases the FinnGen phenotypes included equivalent ICD codes from ICD-8 and/or ICD-9 and/or codes from other diagnostic systems also for inclusion criteria for cases and exclusion criteria for controls, which are not reported here; the codes included here are meant to give the reader an idea as to what the phenotypes encompassed. The authoritative list of diagnostic codes can be found in the FinnGen documentation.

Supplementary Table S2: Heritability estimates for all investigated phenotypes

Trait	Observed-scale h^2	Standard error	p-value
Attention-deficit/hyperactivity disorder	0.0933	0.0043	1.079E-104
Anxiety disorders	0.0763	0.0304	0.00603881
Infections	0.034	0.0084	2.5871E-05
Autoimmune disorders	0.0514	0.0046	2.7358E-29
Malignant neoplasms	0.0225	0.0024	3.4588E-21
Metabolic disorders	0.0203	0.0023	5.4201E-19
Organic mental disorders	0.016	0.0035	2.4221E-06
Cardiovascular diseases	0.0547	0.003	1.4034E-74
Epilepsy	0.0106	0.0021	2.2368E-07
Chronic lower respiratory diseases	0.0543	0.0038	1.2726E-46
Inflammatory bowel diseases	0.0249	0.0027	1.455E-20
Irritable bowel syndrome	0.0094	0.0017	1.6065E-08
Developmental disorders of language, speech, reading and scholastic skills	0.0039	0.0018	0.01513014
Arthropathies	0.0556	0.0027	1.599E-94
Bone and cartilage disorders	0.0105	0.0014	3.1909E-14
Spontaneous abortion	0.0082	0.0031	0.0040826
Complications of labour and delivery	0.0151	0.0026	3.167E-09
Pre-eclampsia or Eclampsia	0.0211	0.003	1.0083E-12
Type 2 diabetes	0.0909	0.0046	3.234E-87
Insomnia	0.0458	0.0021	9.421E-106
Autism spectrum disorder	0.203	0.0154	5.5833E-40
Psychiatric cross-disorder	0.1292	0.0096	1.3753E-41
Obsessive-compulsive disorder	0.3375	0.0475	6.0047E-13
Bipolar disorder	0.0708	0.0027	7.415E-152
Depression	0.0599	0.0023	7.974E-150
Schizophrenia	0.359	0.0117	4.687E-207
Anorexia nervosa	0.1771	0.0121	8.2321E-49
Post-traumatic stress disorder	0.0165	0.003	1.899E-08

Supplementary Table S3: Pairwise genetic correlations for all somatic-psychiatric disorder pairs

Trait 1	Trait 2	r_g	Standard error	p-value	q-value
Attention-deficit/hyperactivity disorder	Arthropathies	0.3514	0.0274	1.19E-37	1.02E-35
Attention-deficit/hyperactivity disorder	Cardiovascular diseases	0.3254	0.0273	9.38E-33	4.01E-31
Attention-deficit/hyperactivity disorder	Chronic lower respiratory diseases	0.3178	0.0283	2.92E-29	8.31E-28
Depression	Arthropathies	0.2852	0.0257	1.29E-28	2.77E-27
Insomnia	Arthropathies	0.3154	0.0297	2.42E-26	4.14E-25
Attention-deficit/hyperactivity disorder	Type 2 diabetes	0.2761	0.0268	6.88E-25	9.81E-24
Depression	Cardiovascular diseases	0.2653	0.0258	8.41E-25	1.03E-23
Depression	Chronic lower respiratory diseases	0.297	0.0299	2.99E-23	3.19E-22
Insomnia	Chronic lower respiratory diseases	0.2773	0.0295	5.46E-21	5.19E-20
Insomnia	Cardiovascular diseases	0.2538	0.0284	4.01E-19	3.43E-18
Psychiatric cross-disorder	Chronic lower respiratory diseases	0.319	0.0379	3.87E-17	3.01E-16
Depression	Metabolic disorders	0.3503	0.0417	4.45E-17	3.17E-16
Depression	Autoimmune disorders	0.2122	0.0256	1.14E-16	7.51E-16
Depression	Irritable bowel syndrome	0.5755	0.0697	1.50E-16	9.14E-16
Psychiatric cross-disorder	Arthropathies	0.2802	0.0356	3.52E-15	2.01E-14
Insomnia	Metabolic disorders	0.3421	0.045	2.91E-14	1.56E-13
Depression	Type 2 diabetes	0.1693	0.0242	2.64E-12	1.27E-11
Psychiatric cross-disorder	Type 2 diabetes	0.2287	0.0327	2.67E-12	1.27E-11
Attention-deficit/hyperactivity disorder	Metabolic disorders	0.3306	0.0483	7.66E-12	3.45E-11
Depression	Bone and cartilage disorders	0.3334	0.0505	4.06E-11	1.74E-10
Attention-deficit/hyperactivity disorder	Irritable bowel syndrome	0.4335	0.066	5.09E-11	2.07E-10
Insomnia	Type 2 diabetes	0.1794	0.0277	9.39E-11	3.65E-10
Insomnia	Irritable bowel syndrome	0.4602	0.0727	2.45E-10	9.11E-10
Attention-deficit/hyperactivity disorder	Infections	0.5603	0.0888	2.80E-10	9.97E-10
Attention-deficit/hyperactivity disorder	Bone and cartilage disorders	0.3174	0.0505	3.28E-10	1.12E-09

Post-traumatic stress disorder	Cardiovascular diseases	0.393	0.0638	7.28E-10	2.40E-09
Psychiatric cross-disorder	Cardiovascular diseases	0.2286	0.0375	1.09E-09	3.45E-09
Insomnia	Bone and cartilage disorders	0.3501	0.0584	2.04E-09	6.22E-09
Post-traumatic stress disorder	Chronic lower respiratory diseases	0.3846	0.0663	6.60E-09	1.95E-08
Post-traumatic stress disorder	Type 2 diabetes	0.3126	0.0546	1.03E-08	2.94E-08
Attention-deficit/hyperactivity disorder	Autoimmune disorders	0.1692	0.0307	3.56E-08	9.82E-08
Attention-deficit/hyperactivity disorder	Epilepsy	0.3363	0.0631	9.84E-08	2.63E-07
Psychiatric cross-disorder	Irritable bowel syndrome	0.4644	0.0883	1.45E-07	3.75E-07
Schizophrenia	Irritable bowel syndrome	0.2265	0.0435	1.92E-07	4.83E-07
Psychiatric cross-disorder	Bone and cartilage disorders	0.3571	0.0693	2.56E-07	6.27E-07
Depression	Epilepsy	0.2687	0.0546	8.60E-07	2.04E-06
Depression	Infections	0.4382	0.0898	1.06E-06	2.46E-06
Psychiatric cross-disorder	Infections	0.5019	0.1038	1.33E-06	2.99E-06
Psychiatric cross-disorder	Metabolic disorders	0.2877	0.0606	2.06E-06	4.52E-06
Schizophrenia	Chronic lower respiratory diseases	0.1152	0.0244	2.34E-06	5.01E-06
Bipolar disorder	Chronic lower respiratory diseases	0.1191	0.0266	7.55E-06	1.58E-05
Schizophrenia	Arthropathies	-0.0962	0.0221	1.34E-05	2.74E-05
Insomnia	Autoimmune disorders	0.1124	0.0266	2.38E-05	4.74E-05
Post-traumatic stress disorder	Arthropathies	0.2372	0.0564	2.60E-05	5.06E-05
Bipolar disorder	Irritable bowel syndrome	0.2312	0.0553	2.90E-05	5.52E-05
Psychiatric cross-disorder	Spontaneous abortion	0.5239	0.1301	5.65E-05	0.000105
Psychiatric cross-disorder	Autoimmune disorders	0.1542	0.039	7.69E-05	0.00014
Psychiatric cross-disorder	Epilepsy	0.2603	0.0731	0.00037	0.000659
Attention-deficit/hyperactivity disorder	Spontaneous abortion	0.4206	0.1215	0.000537	0.000937
Post-traumatic stress disorder	Irritable bowel syndrome	0.4144	0.1201	0.00056	0.000957
Bipolar disorder	Epilepsy	0.1822	0.0529	0.000573	0.000961
Post-traumatic stress disorder	Bone and cartilage disorders	0.3747	0.1094	0.000615	0.001011
Anorexia nervosa	Type 2 diabetes	-0.1152	0.0344	0.000812	0.00131

Developmental disorders of language, speech, reading and scholastic skills	Chronic lower respiratory diseases	0.3843	0.1188	0.001217	0.001928
Depression	Inflammatory bowel diseases	0.0927	0.0291	0.001445	0.002247
Depression	Spontaneous abortion	0.3574	0.1136	0.001654	0.002527
Obsessive-compulsive disorder	Irritable bowel syndrome	0.3587	0.1146	0.001748	0.002623
Bipolar disorder	Spontaneous abortion	0.2764	0.089	0.001899	0.002801
Anxiety disorders	Cardiovascular diseases	0.302	0.1004	0.00263	0.003813
Schizophrenia	Autoimmune disorders	0.069	0.0231	0.002817	0.004017
Schizophrenia	Spontaneous abortion	0.2412	0.0824	0.00342	0.004797
Post-traumatic stress disorder	Epilepsy	0.3695	0.1266	0.003516	0.004851
Insomnia	Epilepsy	0.1582	0.0545	0.003699	0.005023
Depression	Complications of labour and delivery	0.1519	0.0529	0.004086	0.005461
Post-traumatic stress disorder	Metabolic disorders	0.275	0.0974	0.004752	0.006253
Bipolar disorder	Cardiovascular diseases	0.0724	0.0262	0.005721	0.007415
Bipolar disorder	Metabolic disorders	0.1041	0.0381	0.00629	0.007993
Post-traumatic stress disorder	Spontaneous abortion	0.5507	0.2018	0.006354	0.007993
Developmental disorders of language, speech, reading and scholastic skills	Autoimmune disorders	0.2822	0.1046	0.006978	0.008651
Anxiety disorders	Chronic lower respiratory diseases	0.2968	0.1147	0.009664	0.01181
Obsessive-compulsive disorder	Arthropathies	-0.1398	0.0547	0.010596	0.012766
Post-traumatic stress disorder	Autoimmune disorders	0.1672	0.0656	0.01081	0.012844
Developmental disorders of language, speech, reading and scholastic skills	Cardiovascular diseases	0.2708	0.1077	0.011924	0.013973
Bipolar disorder	Type 2 diabetes	0.0627	0.0251	0.012489	0.014438
Developmental disorders of language, speech, reading and scholastic skills	Irritable bowel syndrome	0.6172	0.2479	0.012785	0.014569
Depression	Pre-eclampsia or Eclampsia	0.1101	0.0443	0.012943	0.014569
Anxiety disorders	Arthropathies	0.2317	0.0936	0.013308	0.014784
Autism spectrum disorder	Arthropathies	-0.0924	0.0374	0.013489	0.014794

Post-traumatic stress disorder	Infections	0.3729	0.1525	0.014475	0.015674
Anxiety disorders	Autoimmune disorders	0.234	0.0961	0.014893	0.015925
Developmental disorders of language, speech, reading and scholastic skills	Epilepsy	0.4768	0.2009	0.017629	0.018563
Anorexia nervosa	Complications of labour and delivery	-0.1794	0.0757	0.017794	0.018563
Developmental disorders of language, speech, reading and scholastic skills	Metabolic disorders	0.3517	0.153	0.021522	0.022182
Bipolar disorder	Complications of labour and delivery	0.1203	0.0526	0.022192	0.022589
Autism spectrum disorder	Complications of labour and delivery	-0.1865	0.0817	0.022446	0.022589
Anxiety disorders	Metabolic disorders	0.3149	0.1435	0.028205	0.028055
Insomnia	Infections	0.1689	0.0777	0.029724	0.029227
Post-traumatic stress disorder	Inflammatory bowel diseases	0.157	0.0729	0.031269	0.030396
Anxiety disorders	Type 2 diabetes	0.1799	0.0859	0.036234	0.03459
Attention-deficit/hyperactivity disorder	Pre-eclampsia or Eclampsia	0.1063	0.0508	0.036392	0.03459
Anxiety disorders	Bone and cartilage disorders	0.348	0.1672	0.037403	0.03516
Schizophrenia	Metabolic disorders	0.0657	0.0326	0.043869	0.040791
Post-traumatic stress disorder	Complications of labour and delivery	0.2661	0.1331	0.045581	0.041927
Bipolar disorder	Organic mental disorders	0.1495	0.0772	0.052803	0.048053
Autism spectrum disorder	Irritable bowel syndrome	0.1735	0.0905	0.055222	0.049725
Autism spectrum disorder	Type 2 diabetes	0.0734	0.0392	0.061145	0.054083
Developmental disorders of language, speech, reading and scholastic skills	Organic mental disorders	0.5024	0.2685	0.061326	0.054083
Anorexia nervosa	Spontaneous abortion	0.2333	0.126	0.064085	0.05594
Obsessive-compulsive disorder	Complications of labour and delivery	-0.2084	0.1141	0.06778	0.058567
Obsessive-compulsive disorder	Chronic lower respiratory diseases	-0.088	0.0488	0.071344	0.060501
Anxiety disorders	Complications of labour and delivery	0.4112	0.2281	0.071432	0.060501
Obsessive-compulsive disorder	Inflammatory bowel diseases	0.122	0.0683	0.074061	0.062112
Bipolar disorder	Infections	0.1307	0.0735	0.075366	0.062593
Insomnia	Organic mental disorders	0.142	0.0841	0.091322	0.07504

Schizophrenia	Inflammatory bowel diseases	0.0507	0.0301	0.092107	0.07504
Autism spectrum disorder	Pre-eclampsia or Eclampsia	-0.1209	0.0722	0.094029	0.075577
Insomnia	Inflammatory bowel diseases	0.0632	0.0378	0.094533	0.075577
Schizophrenia	Cardiovascular diseases	0.0399	0.024	0.096413	0.076366
Attention-deficit/hyperactivity disorder	Malignant neoplasms	-0.0645	0.0406	0.112135	0.088004
Attention-deficit/hyperactivity disorder	Organic mental disorders	0.1412	0.0916	0.123199	0.095808
Autism spectrum disorder	Metabolic disorders	0.0873	0.0574	0.128283	0.098864
Schizophrenia	Epilepsy	0.0708	0.0467	0.129504	0.098914
Attention-deficit/hyperactivity disorder	Complications of labour and delivery	0.0947	0.063	0.132794	0.100529
Insomnia	Complications of labour and delivery	0.0893	0.0617	0.147806	0.110911
Anxiety disorders	Irritable bowel syndrome	0.3176	0.2203	0.149395	0.111129
Schizophrenia	Malignant neoplasms	0.0485	0.0349	0.164625	0.121162
Developmental disorders of language, speech, reading and scholastic skills	Malignant neoplasms	-0.1935	0.1396	0.165715	0.121162
Anorexia nervosa	Epilepsy	0.0865	0.0634	0.172456	0.125022
Obsessive-compulsive disorder	Bone and cartilage disorders	-0.1302	0.0969	0.179061	0.128719
Schizophrenia	Type 2 diabetes	0.0287	0.0215	0.181914	0.129681
Anorexia nervosa	Infections	0.1191	0.0896	0.183768	0.12992
Post-traumatic stress disorder	Organic mental disorders	0.183	0.1392	0.188626	0.132261
Developmental disorders of language, speech, reading and scholastic skills	Spontaneous abortion	0.3868	0.3008	0.198476	0.138036
Schizophrenia	Organic mental disorders	0.0687	0.0542	0.204967	0.141401
Autism spectrum disorder	Autoimmune disorders	0.0488	0.0388	0.208489	0.14268
Bipolar disorder	Autoimmune disorders	0.0319	0.0263	0.225157	0.152864
Anxiety disorders	Epilepsy	0.2136	0.182	0.240545	0.162025
Developmental disorders of language, speech, reading and scholastic skills	Inflammatory bowel diseases	0.1468	0.1279	0.251063	0.167789
Depression	Organic mental disorders	0.0624	0.0555	0.260875	0.172633
Obsessive-compulsive disorder	Autoimmune disorders	0.0575	0.0513	0.262348	0.172633

Depression	Malignant neoplasms	-0.0367	0.0334	0.271854	0.177523
Obsessive-compulsive disorder	Type 2 diabetes	-0.0547	0.0508	0.281582	0.182483
Anxiety disorders	Organic mental disorders	0.1848	0.1724	0.283753	0.182507
Post-traumatic stress disorder	Pre-eclampsia or Eclampsia	0.1041	0.0982	0.289108	0.184563
Anxiety disorders	Malignant neoplasms	-0.1436	0.1364	0.292439	0.185307
Insomnia	Spontaneous abortion	0.0805	0.0792	0.309432	0.194633
Obsessive-compulsive disorder	Organic mental disorders	-0.124	0.1246	0.319646	0.19959
Developmental disorders of language, speech, reading and scholastic skills	Type 2 diabetes	0.0894	0.0905	0.323228	0.200364
Obsessive-compulsive disorder	Metabolic disorders	-0.0747	0.0775	0.335111	0.206235
Psychiatric cross-disorder	Pre-eclampsia or Eclampsia	0.0689	0.0735	0.348545	0.212971
Insomnia	Pre-eclampsia or Eclampsia	0.0495	0.0533	0.353042	0.214189
Developmental disorders of language, speech, reading and scholastic skills	Infections	0.2196	0.2423	0.364769	0.219746
Anorexia nervosa	Irritable bowel syndrome	0.0647	0.0718	0.367528	0.219859
Schizophrenia	Infections	0.0562	0.0631	0.373117	0.221652
Obsessive-compulsive disorder	Pre-eclampsia or Eclampsia	0.08	0.0922	0.38557	0.227471
Anorexia nervosa	Bone and cartilage disorders	0.0512	0.0597	0.391102	0.228093
Anxiety disorders	Inflammatory bowel diseases	0.1029	0.1202	0.391957	0.228093
Anorexia nervosa	Chronic lower respiratory diseases	-0.0297	0.0357	0.405447	0.234348
Autism spectrum disorder	Inflammatory bowel diseases	0.0425	0.0523	0.416436	0.239085
Schizophrenia	Pre-eclampsia or Eclampsia	-0.0307	0.041	0.45399	0.25758
Autism spectrum disorder	Malignant neoplasms	-0.0397	0.0531	0.454674	0.25758
Psychiatric cross-disorder	Organic mental disorders	0.0795	0.1091	0.466192	0.262179
Obsessive-compulsive disorder	Infections	-0.0899	0.125	0.472018	0.262179
Anxiety disorders	Infections	0.1581	0.2204	0.473169	0.262179
Schizophrenia	Bone and cartilage disorders	-0.0285	0.0399	0.475051	0.262179
Insomnia	Malignant neoplasms	-0.0235	0.0348	0.499493	0.273902

Bipolar disorder	Pre-eclampsia or Eclampsia	0.0294	0.0466	0.528105	0.286097
Anxiety disorders	Spontaneous abortion	0.2284	0.3623	0.528422	0.286097
Bipolar disorder	Bone and cartilage disorders	0.0272	0.0461	0.555176	0.298688
Psychiatric cross-disorder	Complications of labour and delivery	-0.0455	0.0778	0.55866	0.298688
Attention-deficit/hyperactivity disorder	Inflammatory bowel diseases	0.0202	0.0355	0.569347	0.302511
Bipolar disorder	Arthropathies	0.0129	0.0263	0.623784	0.329389
Developmental disorders of language, speech, reading and scholastic skills	Pre-eclampsia or Eclampsia	-0.0766	0.1695	0.651328	0.341824
Autism spectrum disorder	Cardiovascular diseases	0.0159	0.0415	0.701622	0.365973
Developmental disorders of language, speech, reading and scholastic skills	Complications of labour and delivery	0.0711	0.1929	0.712437	0.366138
Autism spectrum disorder	Chronic lower respiratory diseases	0.0191	0.0522	0.714439	0.366138
Developmental disorders of language, speech, reading and scholastic skills	Arthropathies	0.0349	0.0955	0.714779	0.366138
Post-traumatic stress disorder	Malignant neoplasms	-0.0263	0.0744	0.723718	0.36851
Anorexia nervosa	Metabolic disorders	0.0171	0.0537	0.750154	0.377909
Schizophrenia	Complications of labour and delivery	0.0132	0.0416	0.75101	0.377909
Anorexia nervosa	Cardiovascular diseases	-0.0105	0.0359	0.76992	0.385159
Obsessive-compulsive disorder	Spontaneous abortion	0.0448	0.1595	0.778804	0.387338
Autism spectrum disorder	Epilepsy	-0.0177	0.0726	0.807384	0.392966
Psychiatric cross-disorder	Inflammatory bowel diseases	0.0111	0.0461	0.809725	0.392966
Anorexia nervosa	Pre-eclampsia or Eclampsia	-0.015	0.0642	0.815261	0.392966
Developmental disorders of language, speech, reading and scholastic skills	Bone and cartilage disorders	-0.0382	0.1656	0.817566	0.392966
Anorexia nervosa	Arthropathies	-0.0086	0.038	0.820956	0.392966
Anorexia nervosa	Inflammatory bowel diseases	0.0117	0.0531	0.825607	0.392966
Anorexia nervosa	Malignant neoplasms	-0.0094	0.0433	0.828138	0.392966
Anorexia nervosa	Organic mental disorders	-0.0215	0.0996	0.829094	0.392966
Obsessive-compulsive disorder	Malignant neoplasms	0.0156	0.0733	0.831464	0.392966

Obsessive-compulsive disorder	Epilepsy	-0.0222	0.1074	0.836241	0.393052
Anxiety disorders	Pre-eclampsia or Eclampsia	-0.0326	0.1675	0.845685	0.395319
Anorexia nervosa	Autoimmune disorders	-0.0056	0.0386	0.88465	0.411285
Autism spectrum disorder	Bone and cartilage disorders	-0.0091	0.0677	0.893073	0.412957
Autism spectrum disorder	Spontaneous abortion	0.013	0.1571	0.93405	0.428983
Bipolar disorder	Malignant neoplasms	-0.0031	0.0397	0.93776	0.428983
Psychiatric cross-disorder	Malignant neoplasms	-0.0032	0.0584	0.956302	0.435138
Bipolar disorder	Inflammatory bowel diseases	0.0014	0.0339	0.967058	0.437704
Autism spectrum disorder	Infections	0.003	0.104	0.976987	0.43802
Autism spectrum disorder	Organic mental disorders	0.0027	0.0979	0.977998	0.43802
Obsessive-compulsive disorder	Cardiovascular diseases	0.0005	0.0567	0.992964	0.442407

Supplementary Table S4: Results of the LCV analyses

Trait 1	Trait 2	GCP	p-value	q-value	h^2 warning	r_g warning
Obsessive-compulsive disorder	Irritable bowel syndrome	0.037412	1.01E-63	3.26E-62	no	no
Anxiety disorders	Metabolic disorders	0.400218	1.20E-33	1.94E-32	yes	no
Depression	Spontaneous abortion	-0.83421	7.80E-20	8.40E-19	yes	no
Attention-deficit/hyperactivity disorder	Metabolic disorders	-0.16226	1.67E-18	1.35E-17	no	no
Developmental disorders of language, speech, reading and scholastic skills	Cardiovascular diseases	0.508989	4.34E-14	2.80E-13	yes	no
Post-traumatic stress disorder	Bone and cartilage disorders	0.848351	4.22E-11	2.27E-10	no	no
Anxiety disorders	Bone and cartilage disorders	-0.06732	4.57E-09	2.11E-08	yes	yes
Psychiatric cross-disorder	Infections	-0.83102	9.62E-08	3.88E-07	no	no
Developmental disorders of language, speech, reading and scholastic skills	Chronic lower respiratory diseases	0.706607	3.35E-05	0.000120229	yes	no
Developmental disorders of language, speech, reading and scholastic skills	Irritable bowel syndrome	0.616931	6.83E-05	0.000220611	yes	no
Post-traumatic stress disorder	Epilepsy	0.664785	0.000126	0.000368938	no	no
Post-traumatic stress disorder	Spontaneous abortion	-0.77309	0.000169	0.000455438	yes	no
Post-traumatic stress disorder	Arthropathies	0.360698	0.007479	0.018583141	no	no
Anxiety disorders	Autoimmune disorders	0.624305	0.014334	0.033071478	yes	no
Developmental disorders of language, speech, reading and scholastic skills	Autoimmune disorders	0.625785	0.016913	0.036419316	yes	no
Attention-deficit/hyperactivity disorder	Arthropathies	0.264209	0.018869	0.038091564	no	no
Insomnia	Cardiovascular diseases	0.499703	0.028005	0.053209436	no	no
Attention-deficit/hyperactivity disorder	Irritable bowel syndrome	0.544611	0.066587	0.119487476	no	no
Psychiatric cross-disorder	Chronic lower respiratory diseases	0.552833	0.078439	0.133348591	no	no
Insomnia	Arthropathies	0.250158	0.084469	0.136419121	no	no
Psychiatric cross-disorder	Cardiovascular diseases	0.42263	0.104772	0.156887807	no	no
Depression	Complications of labour and delivery	-0.01402	0.106857	0.156887807	no	no
Depression	Metabolic disorders	0.485307	0.119048	0.163052054	no	no

Depression	Irritable bowel syndrome	-0.47118	0.125485	0.163052054	no	no
Attention-deficit/hyperactivity disorder	Infections	-0.51913	0.1262	0.163052054	no	no
Psychiatric cross-disorder	Metabolic disorders	0.514746	0.13155	0.163426867	no	no
Bipolar disorder	Complications of labour and delivery	-0.5135	0.177273	0.200032642	no	no
Post-traumatic stress disorder	Chronic lower respiratory diseases	0.499087	0.177656	0.200032642	no	no
Depression	Cardiovascular diseases	0.221615	0.1799	0.200032642	no	no
Attention-deficit/hyperactivity disorder	Cardiovascular diseases	0.300584	0.185787	0.200032642	no	no
Attention-deficit/hyperactivity disorder	Pre-eclampsia or Eclampsia	-0.21352	0.216275	0.216153284	no	yes
Psychiatric cross-disorder	Type 2 diabetes	-0.3796	0.219157	0.216153284	no	no
Schizophrenia	Metabolic disorders	-0.29365	0.227199	0.216153284	no	yes
Psychiatric cross-disorder	Bone and cartilage disorders	0.472169	0.230728	0.216153284	no	no
Insomnia	Epilepsy	0.457383	0.246251	0.216153284	no	no
Depression	Pre-eclampsia or Eclampsia	0.057116	0.247186	0.216153284	no	no
Attention-deficit/hyperactivity disorder	Chronic lower respiratory diseases	0.392097	0.252564	0.216153284	no	no
Anxiety disorders	Chronic lower respiratory diseases	0.340505	0.254295	0.216153284	yes	yes
Schizophrenia	Chronic lower respiratory diseases	-0.06354	0.265041	0.219510302	no	no
Insomnia	Bone and cartilage disorders	-0.28802	0.276106	0.222958114	no	no
Bipolar disorder	Chronic lower respiratory diseases	-0.10024	0.290025	0.228485608	no	no
Insomnia	Chronic lower respiratory diseases	0.339709	0.299575	0.230389729	no	no
Bipolar disorder	Organic mental disorders	0.021039	0.319303	0.236536243	yes	yes
Post-traumatic stress disorder	Irritable bowel syndrome	-0.43939	0.333971	0.236536243	no	no
Attention-deficit/hyperactivity disorder	Autoimmune disorders	0.358817	0.335571	0.236536243	no	no
Insomnia	Infections	-0.32009	0.343897	0.236536243	no	no
Anxiety disorders	Type 2 diabetes	0.116196	0.344182	0.236536243	yes	yes
Depression	Infections	-0.26663	0.358428	0.239836649	no	no
Depression	Chronic lower respiratory diseases	0.335234	0.363835	0.239836649	no	no
Depression	Epilepsy	0.438684	0.377634	0.243954296	no	no
Schizophrenia	Irritable bowel syndrome	-0.1906	0.394798	0.246692273	no	no
Post-traumatic stress disorder	Type 2 diabetes	0.369651	0.40122	0.246692273	no	no

Post-traumatic stress disorder	Autoimmune disorders	0.167999	0.408264	0.246692273	no	yes
Depression	Type 2 diabetes	0.416999	0.412422	0.246692273	no	no
Depression	Inflammatory bowel diseases	-0.13566	0.468528	0.275156843	no	no
Autism spectrum disorder	Irritable bowel syndrome	-0.37013	0.491693	0.276637021	no	yes
Post-traumatic stress disorder	Inflammatory bowel diseases	0.363903	0.493354	0.276637021	no	yes
Post-traumatic stress disorder	Infections	-0.31779	0.498615	0.276637021	no	yes
Insomnia	Metabolic disorders	0.22217	0.506043	0.276637021	no	no
Post-traumatic stress disorder	Metabolic disorders	0.394804	0.513871	0.276637021	no	no
Post-traumatic stress disorder	Cardiovascular diseases	0.226103	0.543534	0.287630061	no	no
Bipolar disorder	Irritable bowel syndrome	-0.22051	0.552101	0.287630061	no	no
Schizophrenia	Spontaneous abortion	-0.305	0.565278	0.289820149	yes	no
Developmental disorders of language, speech, reading and scholastic skills	Metabolic disorders	0.338583	0.613495	0.306198603	yes	no
Insomnia	Autoimmune disorders	0.190823	0.616182	0.306198603	no	no
Bipolar disorder	Epilepsy	0.106076	0.628349	0.30751336	no	no
Attention-deficit/hyperactivity disorder	Type 2 diabetes	-0.03321	0.650857	0.309691844	no	no
Bipolar disorder	Type 2 diabetes	0.055182	0.651976	0.309691844	no	no
Psychiatric cross-disorder	Spontaneous abortion	0.142306	0.668419	0.310875783	yes	no
Attention-deficit/hyperactivity disorder	Bone and cartilage disorders	-0.1427	0.687994	0.310875783	no	no
Psychiatric cross-disorder	Epilepsy	0.155082	0.690172	0.310875783	no	no
Attention-deficit/hyperactivity disorder	Spontaneous abortion	-0.25227	0.70023	0.310875783	yes	no
Depression	Autoimmune disorders	0.064319	0.70881	0.310875783	no	no
Schizophrenia	Autoimmune disorders	0.130814	0.717115	0.310875783	no	yes
Psychiatric cross-disorder	Irritable bowel syndrome	-0.21419	0.72184	0.310875783	no	no
Depression	Bone and cartilage disorders	0.248118	0.76456	0.318157961	no	no
Psychiatric cross-disorder	Arthropathies	-0.04836	0.771873	0.318157961	no	no
Anxiety disorders	Arthropathies	0.044012	0.776434	0.318157961	yes	no
Insomnia	Type 2 diabetes	0.276634	0.79113	0.318157961	no	no
Developmental disorders of language, speech, reading and scholastic skills	Epilepsy	0.170558	0.792624	0.318157961	yes	no

Bipolar disorder	Metabolic disorders	0.026554	0.797849	0.318157961	no	no
Bipolar disorder	Spontaneous abortion	0.045575	0.82072	0.323287263	yes	no
Bipolar disorder	Cardiovascular diseases	0.057524	0.842749	0.327965061	no	no
Anxiety disorders	Cardiovascular diseases	0.140754	0.862524	0.331664757	yes	no
Depression	Arthropathies	-0.01714	0.877628	0.333502325	no	no
Post-traumatic stress disorder	Complications of labour and delivery	-0.01669	0.901016	0.338268456	no	yes
Psychiatric cross-disorder	Autoimmune disorders	0.210278	0.911116	0.338268456	no	no
Insomnia	Irritable bowel syndrome	0.068956	0.943299	0.34623725	no	no
Attention-deficit/hyperactivity disorder	Epilepsy	0.077657	0.986386	0.357984524	no	no

GCP: genetic causality proportion; h^2 warning refers to cases in which at least one heritability as estimated by LCV had a z-score below 7; r_g warning refers to cases in which the genetic correlation as estimated by LCV was not significantly different from zero.

Sensitivity analyses for the infections phenotype:

The infections phenotype in iPSYCH was a secondary phenotype. Correcting for the primary phenotype (psychiatric disorders) may lead to an underestimation of the genetic correlation between it and the primary phenotype (because it can lead to an underestimation of specific associations, if the genetic variants is associated with both phenotypes, and both phenotypes are associated with each other), but we repeated the LDSC and LCV analyses for the infections phenotype using a GWAS for infections in which a covariate for any psychiatric diagnosis was included, as a sensitivity analysis.

The heritability was estimated to be 0.0289 (SE=0.0084).

The genetic correlation between the infection phenotype (with the added covariate) and the psychiatric cross-disorder phenotype was 0.3051 (SE=0.1107).

The LCV analysis obtained GCP=-0.8313, $P=3.99\times10^{-11}$, where the psychiatric phenotype was trait 1 and the infections phenotype was trait 2. Note that in this case, the heritability z-score for the infections phenotype was just below 7, at 6.69.
